# miR-200 family controls late steps of postnatal forebrain neurogenesis via Zeb2 inhibition

**DOI:** 10.1038/srep35729

**Published:** 2016-10-21

**Authors:** Christophe Beclin, Philipp Follert, Elke Stappers, Serena Barral, Coré Nathalie, Antoine de Chevigny, Virginie Magnone, Kévin Lebrigand, Ute Bissels, Danny Huylebroeck, Andreas Bosio, Pascal Barbry, Eve Seuntjens, Harold Cremer

**Affiliations:** 1 IBDM, Aix-Marseille Université, CNRS, UMR7288, 13288 Marseille, France; 2 Laboratory of Molecular Biology, Dept Development and Regeneration, KULeuven, 3000 Leuven, Belgium; 3 Miltenyi Biotec GmbH, Bergisch Gladbach, Germany; 4 CNRS and University Nice Sophia Antipolis, IPMC, Sophia Antipolis, France; 5 Dept Cell Biology, Erasmus MC, 3015 CN Rotterdam, The Netherlands; 6 GIGA-Neurosciences, Université de Liège, 4000 Liège, Belgium

## Abstract

During neurogenesis, generation, migration and integration of the correct numbers of each neuron sub-type depends on complex molecular interactions in space and time. MicroRNAs represent a key control level allowing the flexibility and stability needed for this process. Insight into the role of this regulatory pathway in the brain is still limited. We performed a sequential experimental approach using postnatal olfactory bulb neurogenesis in mice, starting from global expression analyses to the investigation of functional interactions between defined microRNAs and their targets. Deep sequencing of small RNAs extracted from defined compartments of the postnatal neurogenic system demonstrated that the miR-200 family is specifically induced during late neuronal differentiation stages. Using *in vivo* strategies we interfered with the entire miR-200 family in loss- and gain-of-function settings, showing a role of miR-200 in neuronal maturation. This function is mediated by targeting the transcription factor Zeb2. Interestingly, so far functional interaction between miR-200 and Zeb2 has been exclusively reported in cancer or cultured stem cells. Our data demonstrate that this regulatory interaction is also active during normal neurogenesis.

Fine-tuning of gene expression is a fundamental requirement for the control of developmental processes. This is particularly evident during nervous system development, where stem cell populations generate a multitude of neuronal and glial cell types in a temporally and quantitatively perfectly orchestrated manner. After their generation, precursors migrate to their respective target structures and form functional connections with their environment. Neurogenesis continues into postnatal and adult stages in defined regions of the mammalian brain, making the control and stabilization of regulatory processes a lifelong requirement^1^. It is evident that complex molecular networks, superposed levels of control and tight interactions between regulatory mechanisms guard induction and maintenance of neurogenesis. MicroRNAs (microRNAs) represent one key control level providing the needed flexibility and stability^2^.

Dicer mutant mouse lines have been widely used to show the general involvement of the microRNA pathway in brain development and function^3^,^4^,^5^,^6^,^7^. Specific microRNAs have been implicated in the control of neurogenesis at different levels. First, they act at the level of initiation of differentiation and the progression of progenitors towards a differentiated state. For example, miR-124 and the miR-9/miR-9* duplex inhibit the expression of molecular components that oppose neuronal differentiation^8^,^9^,^10^,^11^,^12^. Second, they act at the level of neuronal phenotype. This is exemplified by the regulation of dopaminergic fate determination in the forebrain by miR-7a targeting Pax6^13^ or the repartition between inter-neurons and motoneurons in the spinal cord controlled through the targeting of Olig2 by miR-17–3p^14^. Third, microRNAs act at the level of synaptogenesis and synaptic function. For example, miR-134 inhibits dendritogenesis and spine formation^15^,^16^. However, it is likely that additional microRNAs control specific steps of neurogenesis between fate determination at the NSC level and synaptogenesis.

Here we investigate the expression and function of microRNAs during postnatal olfactory bulb (OB) neurogenesis. In this system pre-determined neuronal stem cells in the ventricular/subventricular zone (VZ-SVZ) generate large amounts of neuronal precursors that, after their amplification migrate tangentially within the rostral migratory stream (RMS) into the OB. Once arrived in their target structure they migrate radially into the granular and glomerular layers where they differentiate into interneurons that use GABA, dopamine or glutamate as their neurotransmitters^17^,^18^.

This neurogenic process presents major experimental advantages making it a unique tool for the study of neurobiological problems. First, the process is permanent and not restricted to a small time window in utero. Second, stem cells producing defined neuron populations are regionalized and can be efficiently labeled and manipulated by targeted brain electroporation^19^. Third, different compartments containing cells at distinct stages of the neurogenic process (stem cells, amplifying progenitors, migrating precursors and mature neurons) are spatially separated and can be isolated. Thus, the system is particularly suited to systematically approach the complex regulatory processes that underlie the fine-tuning of neurogenesis by microRNAs. Here, we focus on the role of microRNAs in late steps of neuronal differentiation.

We generated a complete profile of microRNA expression, based on deep sequencing of small RNAs, in the principal compartments of this neurogenic system. Using this unique dataset we identified a family of microRNAs, the miR-200 family, that is specifically expressed at late neurogenic stages but absent from immature differentiation intermediates. We used an *in vivo* approach to perform gain-and loss-of function with the entire miR-200 family leading to promotion or inhibition of neuronal differentiation, respectively. Finally, we show that miR-200 microRNAs function in this context by targeting the zinc-finger transcription factor Zeb2.

## Results

### microRNA expression in the OB neurogenic system: the miR-200 family

We investigated the expression pattern and dynamics of microRNA expression in the OB neurogenic system through the miRNome analysis of defined compartments. We isolated: 1. dorsal and lateral periventricular tissue at P1 (mainly radial glia and transit amplifying cells) and P6 (containing in addition ependymocytes), 2. RMS tissue at P15 (mainly tangentially migrating neuronal precursors and tunnel glia), 3. OB tissue, depleted of the anterior RMS, at P15 and P28 (containing mainly OB neurons and glial cells). Deep sequencing identified 151 microRNAs with an expression level of at least 1000 reads per million (triplicate average) in at least one tissue.

**Figure 1:**
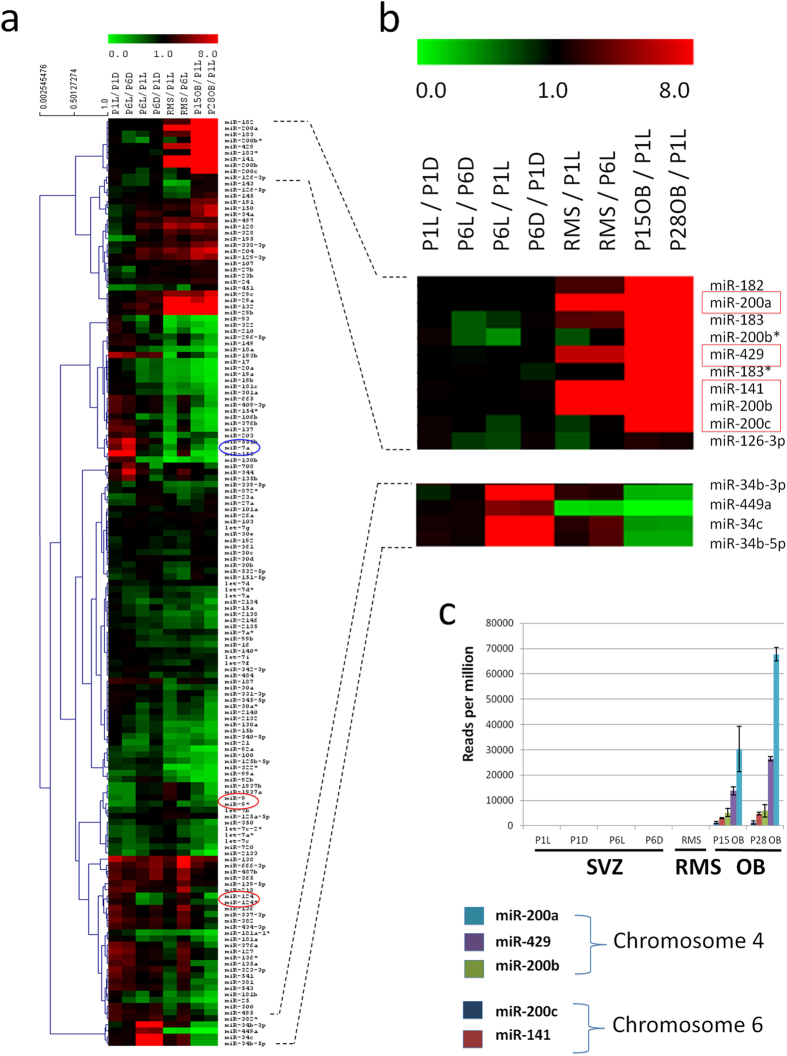
miRNome profiling by deep-sequencing. (**a**) Heatmap showing the results of deep sequencing analyses. Only microRNAs representing more than 0.1% of total microRNAs in at least one tissue are shown. Number of reads for a microRNA in a given tissue was obtained by averaging the different sample repetitions. Columns describe the ratios between tissues selected for comparison. Proximity of vertical position indicates the similarity of expression profile of detected microRNAs and was determined using the MeV application^60^. (**b**) Close-up of specific regions of the heat map highlighting the miR-200-family a group of microRNAs preferentially expressed in the OB and the miR-34 family that is induced during ciliogenesis. miR-34a does not cluster in the heatmap due to strong expression in OB glia. (**c**) Histogram representing the absolute number of reads per tissue obtained in the deep sequencing analyzis for each member of the miR-200 family. All miR-200 family members are exclusively expressed in the OB but not in the stem cell or migratory compartments.

For these 151 miRNAs, we considered the ratios of expression between different samples, thereby comparing spatial and temporal expression in the VZ-SVZ as well as the temporal evolution along the RMS towards the OB. The results, presented as a heat map, are shown in Fig. 1a. Heat map columns represent the different sample combinations selected for comparison. Vertical clustering in the heat map groups miRNA exhibiting similar spatial and temporal expression during the neurogenic process.

Among these 151 microRNAs, some showed stable expression along the neurogenic sequence. This is the case for the neuronal microRNAs miR-9 and miR-124 (red fringes), which maintained high expression in all samples analyzed, in agreement with their general role in the control of the neurogenic sequence progression^20^. Other microRNAs, like for example the pro-dopaminergic microRNAs miR-7a^13^ (blue fringe), showed highly dynamic expression changes both, in space (dorsal vs lateral stem cell compartment) and in time (VZ-SVZ vs OB).

A second interesting observation concerns the behavior of microRNAs families that share common seed sequences. In general, all members of such families show highly similar expression patterns, demonstrated by tight clustering in the heat map, indicating a common function in the system.

This is exemplified by the miR-34/miR-449 family (Fig. 1b). The family is composed of 6 microRNAs, coded by three independent genomic loci. All members play roles in the differentiation of multiciliated cells in several structures^21^,^22^,^23^. We found among the 151 microRNAs expressed above threshold four members, which were all strongly induced in the P6 VZ-SVZ samples compared to P1. This induction parallels the appearance of multiciliated ependymocytes along the ventricular wall during the first postnatal week^24^.

Another family of microRNAs that is tightly regulated during postnatal OB neurogenesis is the miR-200 family, that has been implicated in neurogenesis in cultured cells^25^ and sensory neurons^26^. Indeed, the five members of the miR-200-family were exclusively expressed in the OB and densely clustered in the heat map representation (Fig. 1b,c). In mice, three members (miR-429, miR-200a, miR-200b) reside in one intergenic cluster on chromosome 4. These showed particularly high expression levels (Fig. 1c). miR-200c and miR-141 are localized on chromosome 6 and were expressed at lower levels (Fig. 1c).

**Figure 2:**
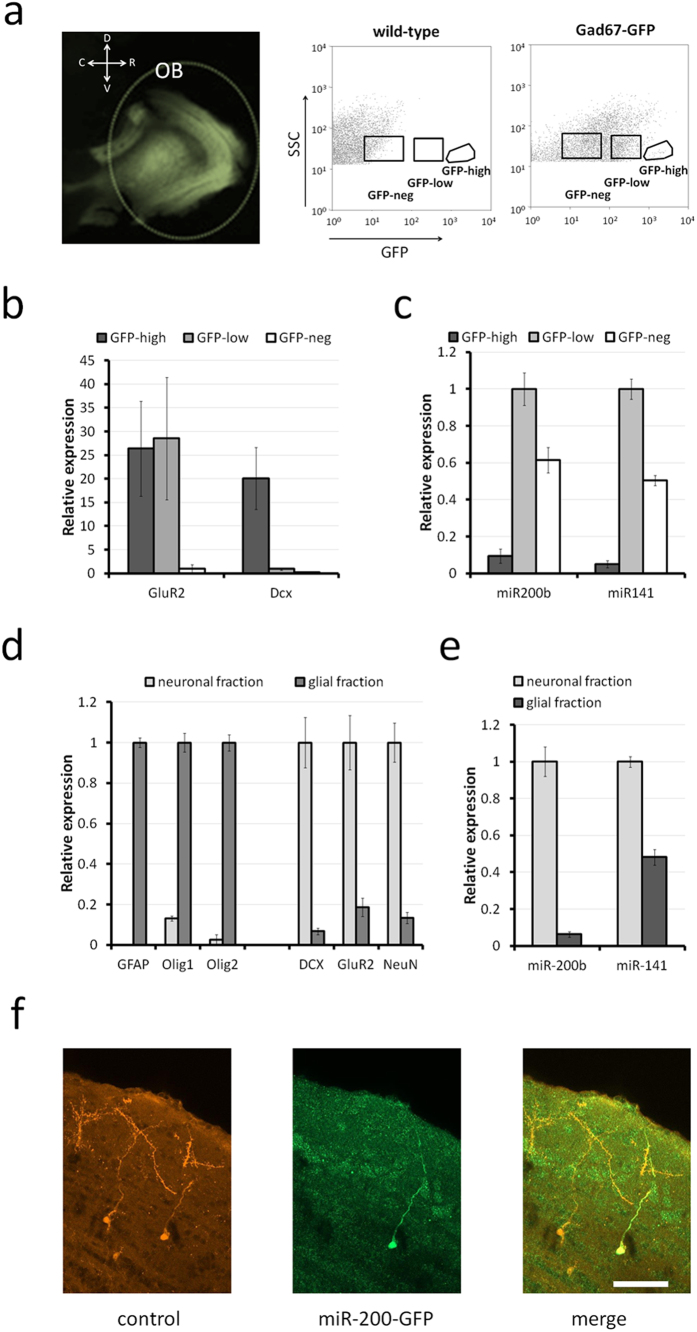
MicroRNA expression in OB subpopulations. (**a**) Sagittal section of a GAD67-GFP knock-in mouse forebrain. Diagrams are dot plots of the FACsorting experiment performed on wild-type (left) and GAD67-GFP knock-in mice (right). Three cell populations were sorted from GAD67-GFP knock-in mouse brain and used for subsequent qRT-PCR analyses. (**b**) qRT-PCR characterization of the three populations. GluR2 is expressed on both, GABAergic neuronal progenitors and fully differentiated neurons. Doublecortin is exclusively expressed in neuronal progenitors. GFP negative cells do not significantly express these neuronal markers. (**c**) qRT-PCR analysis of the expression of miR-200b and miR-141 in the three sorted populations showing a preferential expression in the GFP-low fraction. (**d**) qRT-PCR characterization of the two purified cell fractions issued from the MACS experiment designed to discriminate neuronal vs glial fraction from the OB based. Neuronal (NeuN, GluR2, DCX) and glial (GFAP, Olig1, Olig2) markers validate the expected neuronal and glial identities. (**e**) qRT-PCR analysis demonstrates that miR-200b and miR-141 are enriched in the neuronal fraction. For b-e the qPCR values shown in the histograms result from 2 (**b**,**d**) or 3 (**c**,**e**) qPCR experiments (4 wells per condition in each experiment) (**f**) Electroporation of an expression construct driving GFP with regulatory sequences of the human miR-429/miR-200a/miR-200b cluster leads to GFP-labeled cells in the OB. Scale bar: 70 μm. Error bars: sem.

While micro-dissection before sequencing allowed enriching the samples for the different cell populations of the forebrain neurogenic system, these samples were still heterogeneous, containing, for example, contaminating neurons and glial cells. Therefore we aimed at refining miR-200 family expression in the system combining transgenic and sorting approaches. First, we investigated microRNA expression analyses in the OB neuron sub-populations. In GAD67-GFP knock-in mice the GAD67 promoter drives GFP expression in the GABAergic lineage (Fig. 2a)^27^. We micro-dissected tissue from the OB and performed GFP based FACS sorting after dissociation. These analyses identified three distinguishable cell populations: I) A small population of cells expressing high amounts of GFP (GFP-high). These were positive for the neuron marker GluR2 and the precursor marker Doublecortin (Dcx) demonstrating an immature OB neuron identity (Fig. 2b). II) Cells expressing low amounts of GFP (GFP-low). These expressed GluR2 but not DCX and were therefore likely mature OB neurons. III) Cells that were GFP-negative (GFP-neg; Fig. 2a). These did not express significant levels of either GluR2 or DCX (Fig. 2b), thus likely representing glia and GluR2 negative neurons^28^. qRT-PCR analyses to detect miR-200b and miR-141 as representative members of each of the two miR-200 clusters showed strongest expression in the mature GABAergic (GFP-low) population (Fig. 2c), in agreement with the deep-sequencing data (Fig. 1). This demonstrates that miR-200b and miR-141, and therefore likely the entire miR-200 family, are present in the postnatal neurogenic lineages and that their expression level increases with maturation. The observation that miR-200b and miR-141 are also expressed in the GFP-neg fraction indicates that both micro-RNAs are present either in the glial fraction or in GAD67 negative neurons in the OB, like glutamatergic interneurons or projection neurons such as mitral cells.

To address the latter point, we used Magnetic Activated Cell Sorting (MACS, Miltenyi) to separate neuronal from glial cells after dissociation of the OB of 1-month-old mice and characterized the resulting cell populations by qRT-PCR. As expected, the glial-enriched population expressed high levels of GFAP, Olig1 and Olig2, whereas the neuronal fraction showed strong expression of the neuronal markers NeuN, GluR2 and DCX (Fig. 2d). qRT-PCR analyses demonstrated that miR-200b and miR-141 expression were highest in the neuronal fraction. In the glial fraction miR-200b was expressed at a very low level whereas miR-141 presence was significant (Fig. 2e).

Finally, we introduced the human sequence upstream of the miR-200b/miR-200a/miR-429 cluster^29^ upstream of a GFP-cassette. The resulting plasmid was introduced in neural stem cells in the wall of the forebrain ventricle, together with a control vector (pCAGGS-Tomato), using postnatal brain electroporation^19^. Twenty-four days later a subpopulation of mature granule neurons derived from these transfected stem cells produced both, GFP and tomato proteins, demonstrating activity of the promoter fragment in postnatal generated neurons of the OB (Fig. 2f). Taken together, the above results demonstrate that miR-200 microRNAs expression increases with maturation in the postnatal neuronal lineage that generates OB interneurons.

### miR-200 family microRNAs regulate neuronal differentiation

Next, we investigated the function of the miR-200 family in the control of neuronal differentiation. The miR-200 family contains two different seed sequences (differing in only one nucleotide) and both sequences are present in the two genomic loci. Together with their synchronized expression this suggested a redundant or cooperative function of the miR-200 family members in the OB. Therefore we developed an approach to interfere in parallel with the entire family in both, gain- and loss-of-function settings.

**Figure 3:**
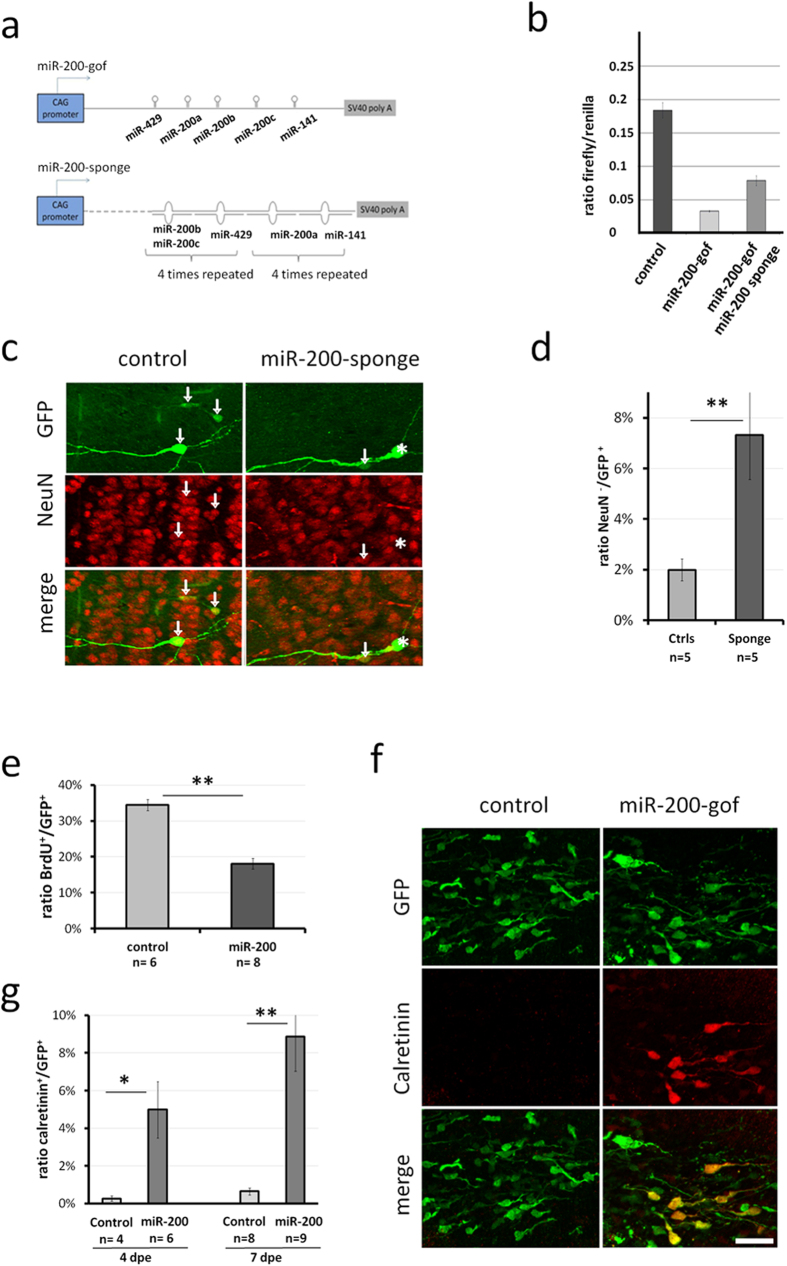
*In vivo* functional analysis of miR-200 microRNAs. (**a**) Representation of the two vectors designed to over-express (miR-200-gof) or down-regulate (miR-200-sponge) the expression of all miR-200 family members in parallel (**b**) Luciferase assay performed on HeLa cells transfected with the Zeb2-UTR vector together with control vectors (control condition), with the miR-200 expression vector alone (miR-200-gof) or with the miR-200 expression vector and the miR-200 sponge plasmid (miR-200-gof + miR-200 sponge condition). Data represent the mean ± s.e.m of values from 4 wells. miR-200 sponge partially rescues the inhibitory activity of the miR-200 expression vector. (**c**) Fluorescent images showing OB neurons 15 days after lateral co-electroporation of pCX-GFP and pCX-mcs2 control vector (left column) or pCX-GFP and miR-200 sponge vector (right column). Arrows indicate cells expressing both GFP and NeuN, asterisk shows a cell positive for GFP but negative for the late pan-neuronal marker NeuN. (**d**) Mean of GFP + cells that do not express NeuN (n = number of animals analyzed). Difference between groups were analyzed using Man and Whitney test (P = 0.009023). (**e**) Ratios of GFP + cells showing BrdU integration 2 days after lateral electroporation of a GFP vector. Difference between groups were analyzed using Man and Whitney test. (**f**) Fluorescent images showing neuroblasts in the RMS 7 days after lateral co-electroporation of pCX-GFP and pCX-mcs2 control vector or pCX-GFP and miR-200-gof stained for calretinin. Only in the miR-200 over-expression condition GFP + cells expressing calretinin are detected. (**g**) Ratios of GFP + cells co-expressing calretinin at 4 and 7 dpe (n = number of animals analyzed). Differences between groups were analyzed using Man and Whitney test. Scale bars: 30 μm in (**c**,**e**).

First, we constructed an *in vivo* expression vector that generates a single transcript containing the two genomic regions harboring the miR-200 family clusters under the control of the chicken β-actin promoter (miR-200-gof, Fig. 3a). Second, we designed a miR-200-sponge that contained four repeats capable to bind each of the miR-200 family members (Fig. 3a).

We validated the constructs using a luciferase assay system. The 3′UTR of the zinc finger/homeodomain transcription factor Zeb2, a well-characterized miR-200 target^30^, was cloned downstream the firefly luciferase gene in the pmiRGlo vector (Promega). Co-transfection of HeLa cells with miR-200-gof and the resulting plasmid strongly repressed luciferase activity. Simultaneous expression of the miR-200-sponge was able to partially restore luciferase activity (Fig. 3b), altogether demonstrating that both vectors were functional. We then used *in vivo* brain electroporation to introduce the miR-200-sponge or miR-200-gof constructs into the OB neurogenic system.

First, we analyzed the consequences of miR-200 inhibition on neuronal differentiation in the OB using the miR-200-sponge. As miR-200 expression occurs during late stages of OB neurogenesis, we analyzed the electroporated cells at 15 dpe, a time point of advanced maturation. Knockdown of miR-200 significantly increased the percentage of electroporated cells in the OB that were negative for NeuN, a marker for mature neurons (control: 1.99% ± 0,43%; miR-200-sponge: 7.32% ± 1.76%; Fig. 3c,d).

Second, we investigated if expression of the miR-200 members at early stages of OB neurogenesis was sufficient to induce premature neuronal differentiation. To this end we electroporated miR-200-gof into the lateral ventricular wall and analyzed their progeny.

A major step in the neurogenic sequence is the transformation of proliferating progenitors into post-mitotic migrating neuroblasts. We thus investigated whether premature expression of miR-200 can induce premature exit of cell-cycle. We measured BrdU incorporation 2 days after electroporation in control and miR-200 gof conditions and found that the percentage of BrdU positive cells was significantly decreased in miR-200 gof conditions (Fig. 3e).

We then investigated the phenotype of electroporated cells in the RMS at 4 days and 7 days post-electroporation. At these stages all GFP-positive cells displayed the typical morphology of migrating neuroblasts (Fig. 3f). In the control situation, calretinin, a late-appearing marker for defined subpopulations of OB interneurons^31^,^32^,^33^,^34^ was absent from the RMS (4 dpe, 0.17% ± 0.17%; 7 dpe, 0.64% ± 0.19, Fig. 3f,g), in accordance with its expression in mature neurons. However, 4 days after miR-200-gof electroporation 4.79% ± 1.15%, (Fig. 3g) of the GFP positive cells in the RMS expressed calretinin and this percentage increased to 8.96%; ± 1.82% at 7 dpe (Fig. 3f,g). Generic markers of differentiation like NeuN and Map2 were unchanged at this time point. At 15 dpe in both, control and miR-200-gof conditions the percentage of GFP + cells expressing calretinin was approximately 2% suggesting that the calretinin expressing neuroblasts at 4 and 7 dpe after miR-200-gof electroporation either downregulated calretinin or died at later stages.

Altogether these results show that knockdown of miR-200 family microRNAs interferes with terminal neuronal differentiation while their premature expression induces in a subset of postnatally generated precursors defined aspects of neuronal maturation: cell-cycle exit and premature expression of a mature neuron marker.

### Mir-200 family target Zeb2 in the OB neurogenic system

**Figure 4:**
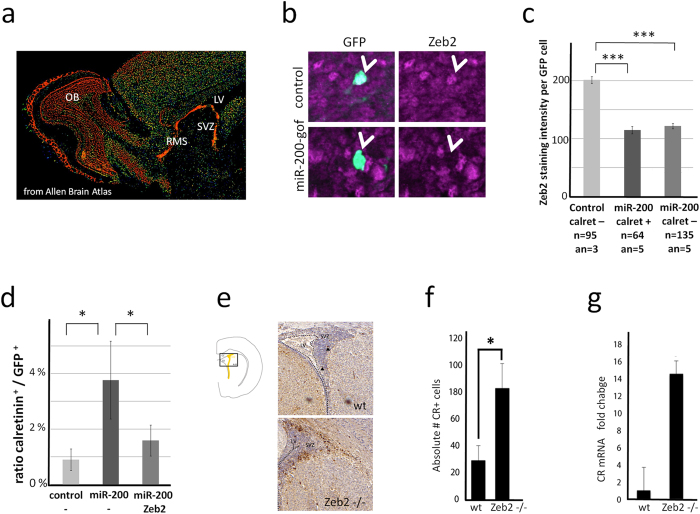
miR-200 induces calretinin expression through Zeb2 inhibition. (**a**) Zeb2 mRNA (red) is widely expressed in the forebrain with particularly prominent presence in the SVZ and RMS. (**b**) Images showing GFP cells in the RMS stained with Zeb2 antibody 4 days after *in vivo* electroporation in control or miR-200-gof conditions. (**c**) Quantification of mean Zeb2 staining intensity 4 days after *in vivo* electroporation cells at 4 dpe in control or miR-200-gof conditions. This showed a significant reduction in Zeb2 protein expression in neuronal precursors, regardless of their calretinin expression status. Differences between groups of cells were analyzed pairwise with a t-test (control vs miR-200 calretinin positive P < 2.2e-16; control vs miR-200 calretinin negative P < 2.2e-16); n = number of cells used for analysis; an = number of animals from which analyzed cells were issued. (**d**) Zeb2 expression normalizes the miR-200-gof mediated induction in calretinin expression. Differences between groups were analyzed pairwise with the Man and Whitney test (control (n = 5 animals) vs miR-200 (n = 5 animals) P = 0.008816, miR-200 (n = 5 animals) vs miR-200 + Zeb2 (n = 7 animals) P = 0.04236). (**e**) Calretinin immunostaining of coronal forebrain section through the SVZ of Gsh2-Cre; Zeb2^+/+^ (wt) or Gsh2-Cre; Zeb^Fl/Fl^ knockout (Zeb2 −/−) animals at P5 at the level indicated in the schema. (**f**) The number of calretinin immunoreactive cells in the aSVZ. is much higher in knockout (Zeb2 −/−) than in control (wt) animals. (**g**) qRT-PCR analysis in FACS sorted SVZ cells from P2 animals reveals a massive increase in calretinin mRNA expression in knockout (Zeb2 −/−) compared to control (wt) animals. In (**f**,**g**) difference between groups was analyzed using t- test. Scale bars: 1 mm in a, 20 μm in (**b**) 200 μm in (**e**).

Next, we aimed at analyzing the regulatory mechanism that underlies the differentiation-inducing function of miR-200 family microRNAs in the system. The best-characterized targets of the miR-200 family, albeit in cancer backgrounds, are the zinc finger proteins Zeb1 and Zeb2^30^. This interaction is a key regulatory mechanism for epithelial-mesenchymal transition, thereby controlling cell migration, stem-cell properties, apoptosis and senescence^30^. *In situ* hybridization data demonstrated that Zeb2 transcripts are particularly strongly expressed in the entire VZ/SVZ-RMS-OB system (Fig. 4a; from Allen brain atlas: http://mouse.brain-map.org). Moreover, immunofluorescence using a Zeb2 specific antibody demonstrated high protein levels in the system (Fig. 4b). We asked if premature expression of miR-200 family microRNAs interfered with Zeb2 levels in neuronal precursors. The miR-200-gof and a GFP- expression vectors were co-electroporated into the SVZ and Zeb2 expression was analyzed in the RMS at 4 dpe. GFP-expressing cells showed significantly less Zeb2 immunoreactivity when miR-200-gof was present (Fig. 4b,c). Moreover, transgenic co-expression of Zeb2 rescued the miR-200 mediated induction in calretinin expression (Fig. 4d). Altogether, these data strongly indicated that the miR-200 induced increase in neuronal differentiation in the RMS was mediated by inhibition of Zeb2.

Finally, we used mouse genetics to further reinforce the link between Zeb2 expression and calretinin induction in postnatal OB neurogenesis. Gsh2-Cre mice target Cre recombinase to SVZ-progenitors that generate neurons for the OB^35^. We crossed Gsh2-Cre mice to *Zeb2*-floxed animals^36^ and analyzed calretinin expression in system. While the periventricular region of control mice was almost devoid of calretinin immunoreactivity at P5, Gsh2Cre/Zeb2^fl^ mice showed significantly increased numbers of calretinin positive cells surrounding the lateral ventricles and extending into the RMS (Fig. 4e,f). We isolated the Cre-targeted cells by FACS of the micro-dissected SVZ at P2 from control and mutant forebrains, and measured the expression level of calretinin by RT-qPCR. The genetic inactivation of *Zeb2* resulted in a more than 14-fold up-regulation of calretinin mRNA (p = 0.0041, n = 6, Fig. 4g), suggesting that it induced the premature maturation of OB interneurons. We conclude that regulation of Zeb2 by miR-200 family microRNAs regulates neuronal maturation during postnatal neurogenesis.

## Discussion

In cancer cells the interaction between miR-200 and Zeb proteins is a key regulatory event in the control of epithelial-mesenchymal transition (EMT) and has been extensively implicated in the metastasis of different cancer types. A role in regulation of developmental processes has been repeatedly proposed, but so far not been demonstrated^30^. Here we show for the first time that this regulatory pathway is active *in vivo* to control a developmental process, the maturation of new neurons.

MicroRNAs appear to be particularly abundant and strongly regulated in the developing and adult brain as concluded from microRNA profiling in crude extracts of total brain tissue or specific structures like cortex at successive embryonic and post-natal stages^37^,^38^,^39^,^40^. Moreover, several specific microRNAs were shown to regulate neurogenesis *in vivo*. For example miR-9 and miR-124 are general regulators of the neurogenic process^12^,^41^ whereas other microRNAs were shown to regulate specific steps during neurogenesis (for review: refs 2 and 42). Finally few microRNAs have been shown to regulate neuronal fate decisions, as exemplified by miR-7a and miR-17-3p^13^,^14^,^43^. However, our understanding of the *in vivo* role of the microRNA pathway in neurogenesis is still limited.

This limitation is mostly due to technical issues, since the particular molecular structure of microRNAs renders traditional approaches difficult. For example, the lack of polyA-tails prevents the use of classical linear amplification protocols and therefore genomic analyses with limited amounts of material. Moreover, the small size and the strong sequence homology between microRNA molecules makes *in situ* hybridization experiments more problematic. Therefore, a global representation of dynamic microRNA expression along neuron differentiation from neural stem cell to mature neurons has, to the best of our knowledge, not been reported.

Here we use the unique features of postnatal OB neurogenesis to investigate the expression of microRNAs during neurogenesis at high resolution. Indeed, during postnatal neurogenesis in the OB, the main neurogenic stages are spatially distinct and can be physically isolated: the VZ-SVZ region contains mitotic progenitors, post-mitotic neuroblasts migrate in the RMS and young neurons terminally differentiate and integrate into the OB circuitry^18^.

Our deep sequencing approach described the dynamics and regionalization of all known microRNAs during the different phases of the forebrain neurogenic process. Among the microRNAs expressed in the system some appeared stably expressed, whereas other are tightly regulated. We focused our functional analysis on the miR-200 family. All members of this family, despite being coded by two independent loci, are induced and function at late stages of the OB neurogenic process.

miR-200 family members are major regulators of tumorigenesis, notably through the capacity to inhibit the transcription factors Zeb1 and Zeb2, two major factors controlling epithelial–mesenchymal-transition^44^,^45^,^46^,^47^. Our immunohistological analyses and *in situ* data (the Allen Brain project) demonstrate strong Zeb2 expression in the forebrain neurogenic system. Moreover, both, Zeb2 loss-of-function and miR-200 gain-of-function led to a comparable phenotype, the premature expression of the late neuronal subtype marker calretinin.

Two alternative explanations can be proposed for this observation. First, repression of Zeb2 by miR-200 microRNAs has a direct impact on differentiation of at least a subfraction of neuronal progenitors. Indeed, in the developing cortex conditional deletion of Zeb2 induced premature neuronal and glial differentiation^48^. Such a role for Zeb2 in the differentiation process would account for the appearance of calretinin positive cells in the RMS in the context of miR-200 overexpression. It would also explain the lack of differentiation, as measured by decreased NeuN staining, when miR-200 is inhibited. Second, induction of calretinin expression in the RMS might be a consequence of slowed neuronal migration. Indeed, it has been shown that interfering with migration through knockdown of DCX leads to the appearance of calretinin positive cells in the RMS^49^. Moreover, direct roles of Zeb2 in in migration of metastatic cancer cells^30^,^50^ and cortical interneurons^36^ have been shown.

Another question concerns the observation that only a small fraction of neuronal precursors shows altered differentiation after interference with miR-200 expression. The quantity of NeuN negative cells in the OB after miR-200 knockdown increases by only 5%, while premature expression of the family induces calretinin in less than 10% of all transfected cells. It should be noted that such minor alterations, typical for the fine-tuning function of microRNAs, would likely be missed in the analysis of other neurogenic processes, which do not permit the same high-resolution analysis. The limited effects might be due to the fact that only a subfraction of the transfected cells are responsive to either inhibition or increase of miR-200 microRNAs. Our finding that the miR-200 promoter fragment that we used to drive GFP expression is only active in a small fraction of the transfected neurons supports this potential lack of competence. Alternatively, it is possible that other microRNAs have redundant functions in the system. In line with this idea, we found that the miR-183/96/182 cluster, a group of microRNAs that has been implicated in the maintenance of retinal neuron integrity^51^,^52^ and shares common predicted targets^53^, appears as nearest neighbors with the miR-200 family in our heat map representation (Fig. 2a,b). Functional studies using tools targeting both groups of microRNAs in the forebrain compartments will be necessary to address this issue.

## Material and Methods

### Mouse lines

Mice carrying floxed *Zeb2* alleles (Zeb2^*fl/fl*^)^54^ were crossed to the RCE reporter mice^55^. Resulting progeny was subsequently crossed with Gsh2-*Cre* mice^56^ to generate Gsh2-Cre; RCE; Zeb2^*fl/fl*^ mutant mice or Gsh2-Cre; RCE; Zeb2^*fl/wt*^ control mice. Animal experiments were carried out in accordance to European Communities Council Directive and approved by French ethical committees (Comité d’Ethique pour l′expérimentation animale n°14; permission number: 62-12112012).

### Plasmid constructs

The pCX-Cre and pCX-GFP vectors are derived from pCX-MCS2^57^. To generate the vector expressing gfp under the control of the human miR-200b/miR-200a/miR-429 regulatory sequence we subcloned gfp from pCX-GFP into the pGL3-1574/ + 120 vector obtained from addgene. The miR-200 expression vector (miR-200-gof) was generated by PCR amplification of both miR-200 clusters from CD1 mouse genomic DNA and sub-cloning of amplified fragments into pCX-MCS2. The sponge construct was designed according to^58^ with 4 repetitions of 2 oligonucleotides (5′-GACACATCGTTACTCTCAGTGTTAGACACGGCATTACTCTCAGTATTA and 5′-GACTTCATCATTACTCCCAGTATTAGACCCATCTTTACTCTCAGTGTTA) partially complementary to any member of the miR-200 family were placed behind a destabilized GFP gene in pCX-d2-GFP plasmid.

Zeb2 3′UTR was PCR-amplified from CD1 mouse brain cDNA and cloned into the pMir-Glo vector (Promega) to generate the 3′UTR-Zeb2 pmiRGlo vector.

### RNA extraction and deep sequencing

Total RNA was extracted from CD1 mice using the miRNeasy kit (Qiagen). RNA was extracted from dorsal or lateral VZ-SVZ at P1 and P6, from RMS at P15 and P28 and from OB at P15 and P28. All samples were dissected in triplicate. Deep sequencing analysis were performed on the 15–50 bp RNA molecules using the Applied Biosystems SOLiD™ System. For each sample, results were normalized for each microRNA as number of reads per million. Results were submitted to GEO (GSE60817).

### Cell sorting (FACS, MACS), qRT-PCR and luciferase assay

For isolation of OB interneurons from GAD67-GFP knock-in mice^59^, whole bulbs of P30 animals were dissected and dissociated by Trypsin/DNAse digestion. GFP cells were purified using MoFlow (Beckman-Coulter) flow cytometer. For Zeb2 mutant analyses, lateral SVZ of P2 control brains were dissected and dissociated by Papain (Sigma)/DNAse digestion. GFP Cells (provided by the RCE locus) were sorted using a FACSAriaI (BD Biosciences).

To separate neuronal from glial cells by MACS, OB from P30 CD1 mice were subjected to Trypsin/DNAse dissociation. Both neuronal and glial enriched fractions were recovered from single cell suspension containing approximately 1 × 10^6^ cells using the “Neuron Isolation Kit” (Miltenyi).

RNAs were extracted from sorted cells using the mRNeasy or miRNeasy kit (Qiagen). cDNAs were prepared using superscript-III (Life-Technologies) and qPCR was performed using SYBR-GreenER qPCR SuperMix (Life-Technologies), except for Zeb2KO sorted cells which were performed on a LightCycler 480 Instrument (Roche) using SYBR Green PCR Master Mix (Roche). Beta-Actin was used as reference gene. Primers sequences are given in SI. MicroRNA qPCR was performed using LNA-qRT-PCR system from Exiqon and using U6 as reference gene.

Luciferase assay was performed on HeLa cells 48 h after Lipofectamine 2000 (Life-Technologies) mediated transfection using the Dual-Luciferase Reporter Assay (Promega) and a Luminometer (Berthold Technologies).

### Immunohistochemistry and Image analysis

Brain sections and staining experiments were performed as in^19^ except those performed on Gsh2-Cre; RCE; Zeb2^*fl/fl*^ mice brains processed as in^48^. Primary antibodies used are: Calretinin (rabbit, Swant, 1/1000), GFP (chicken, Aves, 1/500), mouse IgG1 anti-NeuN (Millipore, 1:100), rat Igg2a anti-BrdU (AbD Serotec (Oxford B), 1/1000). Images were taken using a fluorescence microscope (Axiolmager Z1, ApoTome system, Zeiss) except for Gsh2-Cre; RCE; Zeb2^*fl/fl*^ sections (Leica DMR microscope) and for spine density measurement (laser confocal scanning microscope, LSM510, Zeiss - magnification: 63x). Data in graphics are presented as mean ± s.e.m of values obtained on n samples (*P < 0,05. **P < 0,01, ***P < 0,001). For BrdU incorporation analysis, animals at 2 dpe were injected once with a BrdU solution (50 μg/g body weight, Sigma, Saint-Louis MO) 2 hours before perfusion. BrdU staining was performed after 15 min incubation at 37° in 2N HCl-0.5%. In Fig. 4c, Zeb2 expression level per transfected cell was assessed as follows. Transfected cells in the RMS were identified based on GFP expression. Quantification of Zeb2 staining was performed using ImageJ software on a single z-plan focused on the nucleus (chosen using DAPI staining). A ROI was subsequently drawn inside the nucleus area and the mean intensity of Zeb2 staining signal was then measured across the ROI.

## Additional Information

**How to cite this article**: Beclin, C. *et al*. miR-200 family controls late steps of postnatal forebrain neurogenesis via Zeb2 inhibition. *Sci. Rep.* **6**, 35729; doi: 10.1038/srep35729 (2016).
