# Corrigendum: miR-200 family controls late steps of postnatal forebrain neurogenesis via Zeb2 inhibition

**DOI:** 10.1038/srep39368

**Published:** 2016-12-21

**Authors:** Christophe Beclin, Philipp Follert, Elke Stappers, Serena Barral, Nathalie Coré, Antoine de Chevigny, Virginie Magnone, Kévin Lebrigand, Ute Bissels, Danny Huylebroeck, Andreas Bosio, Pascal Barbry, Eve Seuntjens, Harold Cremer

Scientific Reports 6: Article number: 35729; 10.1038/srep35729 published online: 10 21 2016; updated: 12 21 2016.

The original version of this Article contained a typographical error in the spelling of the author Nathalie Coré, which was incorrectly given as Coré Nathalie. This has now been corrected in the PDF and HTML versions of the Article.

